# Therapeutic versus neuroinflammatory effects of passive immunization is dependent on Aβ/amyloid burden in a transgenic mouse model of Alzheimer's disease

**DOI:** 10.1186/1742-2094-7-57

**Published:** 2010-09-28

**Authors:** S Sakura Minami, Elkhansa Sidahmed, Saba Aid, Mika Shimoji, Takako Niikura, Italo Mocchetti, G William Rebeck, Jay S Prendergast, Chris Dealwis, Ronald Wetzel, Francesca Bosetti, Yasuji Matsuoka, Hyang-Sook Hoe, R Scott Turner

**Affiliations:** 1 Department of Neuroscience, Georgetown University Medical Center, 3970 Reservoir Road NW, Washington, DC 20057, USA; 2 Department of Neurology, Bldg. D, Suite 177, Georgetown University Medical Center, 4000 Reservoir Road NW, Washington, DC 20057, USA; 3 National Institutes of Health, Bethesda, MD, USA; 4 Department of Pharmacology, Case Western Reserve University, 10900 Euclid Ave., Cleveland, OH 44106, USA; 5 Structural Biology Department and Pittsburgh Institute for Neurodegenerative Diseases, Pittsburgh, PA 15260, USA

## Abstract

**Background:**

Passive immunization with antibodies directed to Aβ decreases brain Aβ/amyloid burden and preserves memory in transgenic mouse models of Alzheimer's disease (AD). This therapeutic strategy is under intense scrutiny in clinical studies, but its application is limited by neuroinflammatory side effects (autoimmune encephalitis and vasogenic edema).

**Methods:**

We intravenously administered the monoclonal Aβ protofibril antibody PFA1 to aged (22 month) male and female 3 × tg AD mice with intermediate or advanced AD-like neuropathologies, respectively, and measured brain and serum Aβ and CNS cytokine levels. We also examined 17 month old 3 × tg AD female mice with intermediate pathology to determine the effect of amyloid burden on responses to passive immunization.

**Results:**

The 22 month old male mice immunized with PFA1 had decreased brain Aβ, increased serum Aβ, and no change in CNS cytokine levels. In contrast, 22 month old immunized female mice revealed no change in brain Aβ, decreased serum Aβ, and increased CNS cytokine levels. Identical experiments in younger (17 month old) female 3 × tg AD mice with intermediate AD-like neuropathologies revealed a trend towards decreased brain Aβ and increased serum Aβ accompanied by a decrease in CNS MCP-1.

**Conclusions:**

These data suggest that passive immunization with PFA1 in 3 × tg AD mice with intermediate disease burden, regardless of sex, is effective in mediating potentially therapeutic effects such as lowering brain Aβ. In contrast, passive immunization of mice with a more advanced amyloid burden may result in potentially adverse effects (encephalitis and vasogenic edema) mediated by certain proinflammatory cytokines.

## Background

A defining pathological hallmark of Alzheimer's disease (AD) is the accumulation of Aβ/amyloid deposits in brain. The generation and clearance of Aβ, produced from amyloid precursor protein (APP) by β- and γ-secretases, remain key therapeutic targets for transgenic AD mouse models and for clinical studies [1]. Aβ naturally exists as a monomer or as aggregates including oligomers, protofibrils, and fibrils. Of the two most commonly generated isoforms of Aβ, Aβ40 and Aβ42, the latter aggregates more readily to form amyloid fibrils [2,3]. Oligomers and protofibrils, the intermediate stages of Aβ fibril formation, are neurotoxic in culture and in animal models [4-6] and these aggregates may correlate better with AD severity than neuritic plaque density [7,8].

Immunotherapy is an effective method of reducing brain Aβ levels and preserving or improving behavioral outcome measures in transgenic mouse models of AD. Initial studies utilized an active immunization approach. Peripheral injections of synthetic human Aβ prevented amyloid deposition, decreased CNS Aβ/amyloid burden, and ameliorated memory deficits in mice [9-11]. However, when this therapy was translated to individuals with AD, the pivotal clinical trial was terminated early due to the development of excessive neuroinflammation (autoimmune encephalitis) in 6% of treated individuals [12]. Passive immunization with antibodies to Aβ may offer a safer, and reversible, alternative by circumventing T-cell responses associated with neuroinflammation in active immunization protocols. Passive immunization studies show similar efficacy in reducing brain Aβ/amyloid and preserving memory in transgenic mouse models of AD [13,14], but the passive immunization approach is also limited by excessive neuroinflammation and vasogenic edema in a subset of treated individuals with AD.

Passive immunization may reduce brain Aβ by at least two distinct mechanisms that are not mutually exclusive: 1) microglial phagocytosis of Aβ/amyloid with increased cytokine production, which requires peripherally administered antibodies to cross the blood brain barrier [13], and 2) peripheral sequestration of Aβ by antibody in the blood -- referred to as the sink hypothesis [14]. The Aβ antibody in the periphery may act as a sink by binding Aβ in the blood and promoting clearance of Aβ from the brain to the periphery, resulting in increased plasma Aβ following treatment. Others suggest, however, that peripherally-administered antibody also prolongs the half-life of Aβ in blood, and this explains the resultant increase in plasma levels. To examine these possibilities, it is important to measure CNS cytokine levels in addition to serum and brain Aβ levels following passive immunization. It is unknown whether immunotherapy may prove beneficial with high levels of CNS amyloid burden, or whether treatment may perhaps be limited to a subset of individuals in earlier disease stages. Alternatively, antibody dosage may be tailored to amyloid burden in order to minimize the risk of encephalitis and vasogenic edema. A recent clinical study reported increased vasogenic edema with higher antibody dosage and greater disease burden (as found in ApoE4-positive subjects) [15]. This is the first study to suggest differential responses to passive immunization perhaps due to CNS amyloid burden in a transgenic mouse model of AD.

We examined whether passive immunization of 3 × tg AD mice with the monoclonal Aβ protofibril antibody PFA1, which is well-characterized but has not yet been tested as an immunotherapeutic [16,17], was effective in lowering brain Aβ. The use of 3 × tg AD mice (which express three human mutant transgenes--APP, PS-1, and Tau--found in familial AD or a familial tauopathy) allowed us to investigate CNS Aβ levels as well as downstream effects of PFA1 immunization on tau phosphorylation. The 3 × tg AD mice are also known to have sex-specific differences in CNS amyloid burden, where females develop Aβ pathology at an earlier age and to a much greater extent than males [18]. This allowed us to investigate the potential roles of sex and amyloid burden in response to passive immunotherapy. Passive immunization of intermediate but not more advanced stage 3 × tg AD mice resulted in decreased brain Aβ and increased serum Aβ, supporting the sink hypothesis. In the older female mice with advanced pathology we found no change in brain Aβ and an increase in CNS proinflammatory cytokine levels. Collectively, these data suggest that the potential benefits of passive (and active) immunization may be optimal in individuals with less pathology, and that treatment of individuals with more advanced CNS amyloid burden may engender potentially deleterious neuroinflammation. Passive (and active) immunization therapies may require tailoring to Aβ/amyloid disease burden, and may be considered high risk in subpopulations with advanced disease due to the development of neuroinflammation, encephalitis, and vasogenic edema. The critical mediators of neuroinflammation in response to passive immunization in tg AD mice or in man, however, remain unclear.

## Methods

### Animals

We obtained 3 × tg AD mice (breeding pairs) from Dr. Frank LaFerla (University of California, Irvine) to establish our colony. These mice were generated by co-microinjection of mutant APP (K670M/N671L) and tau (P301L) transgenes under the control of the Thy 1.2 promoter into mutant PS-1 (M146V) knock-in mice [19]. We used 3 × tg AD mice at 21.6 ± 0.5 months of age for vehicle (PBS)-treated females (n = 6), PFA1-treated females (n = 7), vehicle-treated males (n = 5), and PFA1-treated males (n = 7). We also used 3 × tg AD mice at 16.8 ± 0.6 months of age for vehicle-treated females (n = 5) and PFA1-treated females (n = 5). Mice were sacrificed by cervical dislocation to eliminate anesthesia-mediated tau phosphorylation [20], and brains were snap-frozen in dry ice for biochemical analyses. All animal experiments were approved by the Institutional Animal Care and Use Committee at Georgetown University.

### Antibodies and chemicals

The protofibril Aβ monoclonal antibody PFA1 was purified as described [16,17]. The monoclonal antibody 6E10 was purchased from Invitrogen (Carlsbad, CA). Phospho-tau antibodies AT8 (Ser202/Thr205) and AT180 (Thr231) were purchased from Pierce Biotechnology (Rockford, IL), and Tau46 detecting total tau was purchased from Sigma-Aldrich (St. Louis, MO). Synthetic Aβ40 and Aβ42 were purchased from American Peptide (Sunnyvale, CA) and a fraction was dissolved to a concentration of 0.1 μg/μL ddH_2_0 for gel loading for immunoblots.

### Mouse treatments and plasma collection

Purified PFA1 (100 μg IgG/mouse) or vehicle control (PBS) was intravenously administered into the tail vein weekly for 4 weeks. Blood was collected from the facial vein 1 day prior to the first infusion and from the heart 1 week following the last injection -- at the time of sacrifice. EDTA-treated plasma was prepared, aliquoted, snap frozen, and stored at -80°C for biochemical assays.

### Sample preparation and Aβ ELISAs

Brains were homogenized in a 10-fold volume of 50 mM Tris-HCl buffer, pH 7.6, containing 250 mM sucrose and protease and phosphatase inhibitors. This crude brain homogenate was used for immunoblot analysis.

Proteins for immunoblot analysis of tau were separated on a 4-15% gradient Tris-HCl SDS-PAGE gel and transferred to a PVDF membrane. Aβ samples were separated on a 10-20% gradient Tris-Tricine gel (BioRad, Hercules, CA). The membrane was blocked with 5% non-fat dry milk and probed with the appropriate primary antibody and HRP-coupled anti-mouse IgG secondary antibody (Jackson ImmunoResearch, West Grove, PA). Bands were visualized using a chemiluminescence kit (Pierce, Rockford, IL) and densitometrically quantified (Quantity One, BioRad).

Soluble Aβ was extracted in 0.4% diethylamine (DEA) as previously described [21]. Briefly, crude 10% homogenate was mixed with an equal volume of 0.4% DEA, sonicated, and ultracentrifuged for 1 hour at 100,000 × g. The supernatant was collected and neutralized with 10% 0.5 M Tris base, pH 6.8. The resulting DEA fraction was used for soluble Aβ analyses. For analysis of 22 month old mice, total Aβ was analyzed from 10% crude brain homogenate mixed with 70% FA, which was then sonicated and ultracentrifuged for 1 hour at 100,000 × g. The insoluble fraction was calculated as the difference between total and soluble Aβ. For analysis of 17 month old mice, we undertook a more direct approach for measurement of insoluble Aβ by resuspending the remaining pellet following DEA fractionation in 70% FA, sonicating, and ultracentrifuging to yield the insoluble Aβ fraction. Total Aβ was calculated as the sum of measured soluble and insoluble Aβ. Sensitive and specific ELISAs to human Aβ1-40 and Aβ1-42 were purchased from IBL Transatlantic (Toronto, Canada) and conducted per the manufacturer's protocol.

### Cytokine ELISAs

Levels of inflammatory cytokines IL1β, TGFβ, TNFα, MCP-1, and IL4 were measured using Multiplex ELISA assays from Search Light Sample Testing Service (Pierce). Levels of SDF1 and RANTES were measured using ELISA kits from R & D Systems (Minneapolis, MN).

### Statistical analyses

Immunoblots for PFA1 characterization were performed a minimum of three times. Immunoblots for phospho-tau and total tau quantification were performed a minimum of two times for all samples collected. All statistics were calculated using GraphPad Prism 5 software, using student's t-test with significance determined as p < 0.05. Descriptive statistics are displayed as mean ± S.E.M. Correlational analyses were performed on pairs of variables and the correlation is indicated as an R^2 ^value within a 95% confidence interval, with p < 0.05.

## Results

### PFA1 detects Aβ monomers and oligomers

**Figure 1:**
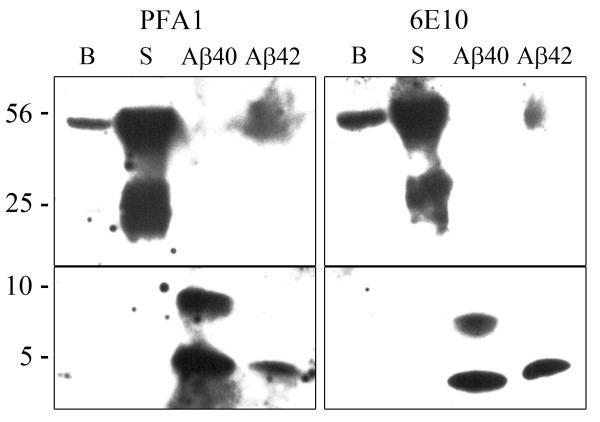
**The protofibril monoclonal antibody PFA1 recognizes multiple Aβ species**. 5 μL of serum (S) and 10 μg of soluble protein extract from brains (B) of 22 month old AD 3 × tg mice were immunoblotted for Aβ with antibody PFA1 (left) or 6E10 (right). 1 μM synthetic Aβ40 and Aβ42 (right lanes) were loaded for comparison. Membranes were cut at 15 kDa to subject the lower panels to a longer exposure for detection purposes. PFA1 detected the 56 kDa oligomeric form of Aβ from serum with high affinity, similar to 6E10.

The monoclonal PFA1 antibody was designed and characterized to detect various forms of Aβ [16]. To determine which Aβ species from AD 3 × tg mice are detected by PFA1, we performed an immunoblot of serum proteins and proteins extracted from brain homogenates of 3 × tg AD mice, with pure synthetic human Aβ40 and Aβ42 as positive controls. PFA1 (left panel) detected monomeric Aβ40 and Aβ42; however, PFA1 detected oligomeric forms of Aβ42 with apparent higher affinity than Aβ40 (Fig. 1). The major Aβ species recognized by PFA1 in serum and in brain homogenates was the 56 kDa complex Aβ*56, most likely a tetramer of Aβ trimers thought to be the leading culprit inducing memory deficits in an animal model of AD [6]. The monoclonal antibody 6E10 (right panel) similarly recognized multiple species of Aβ -- comparable to those detected by PFA1. Membranes probed with both PFA1 (left) and 6E10 (right) were cut at ~15 kDa so that the lower panels could be subjected to longer exposures. These studies demonstrate that the PFA1 antibody detects both monomeric and oligomeric forms of Aβ derived from 3 × tg AD mice.

### Total brain Aβ is decreased in immunized 22 month old male mice

**Figure 2:**
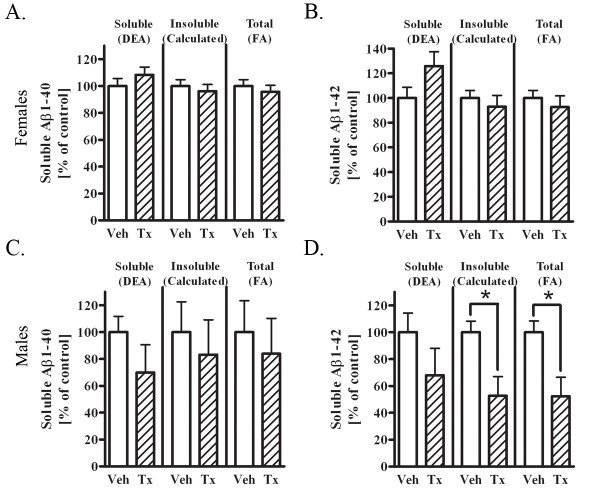
**Brain Aβ1-42 is decreased in 22 month-old PFA1 treated male mice**. A. Aβ1-40 levels were measured from DEA-extracted (soluble proteins, left) and FA-extracted (total proteins, right) brain lysates from vehicle (n = 6) or PFA1-treated (n = 7) female mice following 4 weeks of treatment. Insoluble Aβ levels were calculated as the difference between total and soluble Aβ. There were no significant differences in soluble, insoluble, or total Aβ1-40 levels between vehicle and PFA1-treated female mice. B. Aβ1-42 levels were measured from DEA and FA lysates from the same female mice treated with vehicle or PFA1. There were no significant differences in Aβ1-42 between vehicle and PFA1-treated female mice. C. Aβ1-40 levels were measured from DEA and FA lysates from vehicle (n = 5) or PFA1-treated (n = 7) male mice. There were trends towards decreased soluble (30%), insoluble (16%) and total (17%) Aβ1-40 in PFA1-treated mice. D. Aβ1-42 levels were measured from DEA and FA lysates from vehicle or PFA1-treated male mice following 4 weeks of treatment. PFA1-treated mice exhibited a trend towards decreased soluble Aβ1-42 (32%) and had a significant decrease in insoluble and total Aβ1-42 (48%, p < 0.05, and 47%, p < 0.05, respectively).

To determine whether passive immunization of aged 3 × tg AD mice decreased brain Aβ, we administered weekly intravenous injections of PFA1 (100 μg/mouse/week) to 22 month-old male and female mice for four weeks. Brain homogenates were analyzed for DEA-soluble and total (FA extracted) Aβ40 and Aβ42. Insoluble Aβ was calculated as the difference between total and soluble Aβ. We found no significant differences in soluble, insoluble, or total Aβ40 and Aβ42 in female mice immunized with PFA1 (Fig. 2A &2B). In contrast, we found a significant 48% decrease in insoluble Aβ42, a significant 47% decrease in total Aβ42, and a downward trend in soluble Aβ42 (32%) following treatment of immunized male mice at the same age (Fig. 2D). We found similar trends of decreased Aβ40, in both the soluble (30%) and insoluble (16%) fractions from male mice (Fig. 2C). Relative values between females and males for Aβ40 and Aβ42 were as expected, with females showing higher levels of Aβ40 (60-70 pmol/g tissue) compared to males (15-20 pmol/g tissue) and higher levels of Aβ42 (30-40 pmol/g tissue) compared to males (9-14 pmol/g tissue). These data reveal that passive immunization with PFA1 decreased brain Aβ levels in male, but not female, 22 month-old 3 × tg AD mice.

### Plasma Aβ is increased in immunized 22 month old male mice

**Figure 3:**
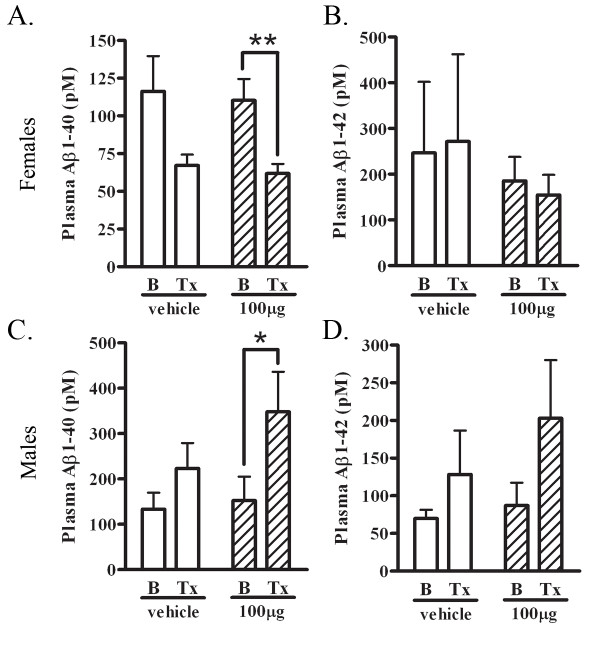
**Plasma Aβ is decreased in 22 month old PFA1 treated female mice and increased in 22 month old PFA1 treated male mice**. A. Aβ1-40 levels were measured from plasma collected from vehicle and PFA1 treated female mice before and after 4 weeks of treatment. There was no significant change in plasma Aβ1-40 levels following treatment in vehicle treated animals. There was a significant 44% decrease (p < 0.01) in plasma Aβ1-40 levels following PFA1 treatment. B. Aβ1-42 levels were measured from plasma collected from vehicle and PFA1 treated female mice before and after treatment. There were no significant changes to Aβ1-42 in either the vehicle or PFA1 group following treatment. C. Aβ1-40 levels were measured from plasma collected from vehicle and PFA1 treated male mice before and after 4 weeks of treatment. There was no significant change in plasma Aβ1-40 levels following treatment in vehicle treated male mice. There was a significant 128% increase (p < 0.05) in Aβ1-40 levels following treatment in the PFA1 treated male mice. D. Aβ1-42 levels were measured from plasma collected from male mice before and after vehicle or PFA1 treatment. There were no significant changes to Aβ1-42 levels following treatment in either vehicle or PFA1 group.

Plasma Aβ levels were measured before and after the 4 week treatment paradigm to determine whether PFA1 sequestered Aβ in the periphery. There was a significant 44% decrease (p < 0.01) in plasma Aβ40 levels in 22 month-old immunized female mice, and no differences in plasma Aβ42 levels following treatment (Fig. 3A and 3B). However, there was a significant 128% increase (p < 0.05) in plasma Aβ40 in males after treatment (Fig. 3C). Male mice also showed a similar increasing but non-significant trend (133%) in plasma Aβ42 following treatment (Fig. 3D). These data provide evidence for peripheral Aβ sequestration in 22 month-old male mice through a decrease in brain Aβ with a corresponding increase in plasma Aβ.

### CNS TNFα and MCP-1 levels are increased in immunized 22 month old female mice, and unchanged in 22 month old male mice

**Figure 4:**
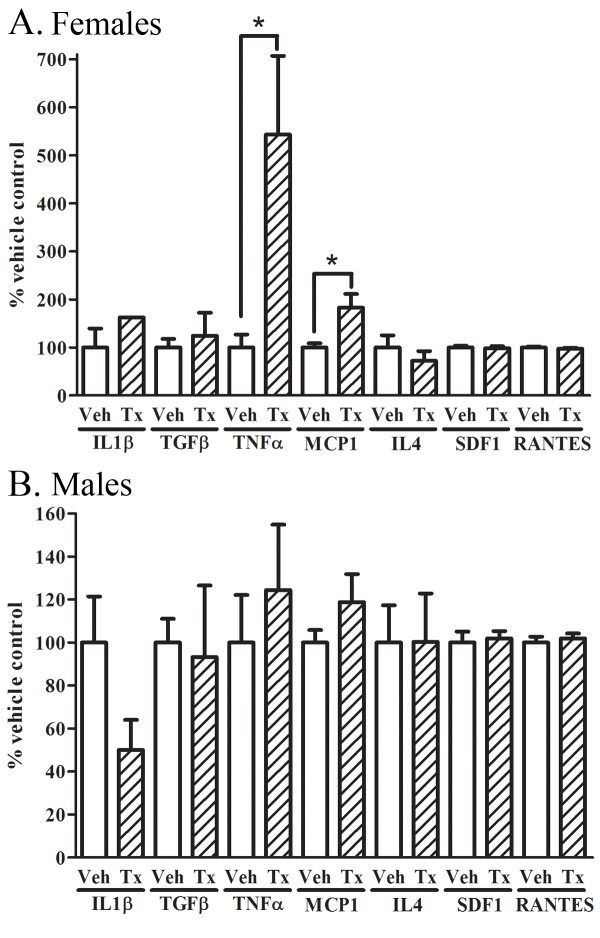
**CNS TNFα and MCP-1 levels are increased in 22 month old immunized female mice and unchanged in male mice**. A. Levels of inflammatory cytokines IL1β, TGFβ, TNFα, MCP-1, IL4, SDF1, and RANTES were measured from brain homogenates of vehicle or PFA1 treated female mice. There were no significant differences in IL1β, TGFβ, IL4, SDF1, or RANTES between vehicle and PFA1 treated groups. There was a significant 459% (p < 0.05) increase in TNFα, and an 83% increase (p < 0.05) in MCP-1 following PFA1 treatment in female mice. B. Levels of inflammatory cytokines IL1β, TGFβ, TNFα, MCP-1, IL4, SDF1, and RANTES were measured from brain homogenates of vehicle or PFA1 treated male mice. There were no significant differences in any of the cytokines assayed.

Passive immunization to Aβ/amyloid may activate microglia since a small fraction of antibody delivered intravenously may penetrate the blood-brain barrier. To determine whether passive immunization induced neuroinflammatory responses, we examined cytokines and chemokines in brain homogenates of 22 month old 3 × tg AD mice following four weeks of treatment, including Interleukin-1β (IL-1β), Transforming Growth Factor β (TGFβ), Tumor Necrosis Factor α (TNFα), Monocyte Chemotactic Protein-1 (MCP-1, now CCL2, Chemokine (C-C) motif ligand 2), Interleukin-4 (IL-4), stromal cell-derived factor-1 (SDF1), and Chemokine (C-C) motif ligand 5 (CCL5, or RANTES). Immunized females exhibited markedly increased levels of CNS TNFα (by 459%, p < 0.05) and MCP-1 (by 83%, p < 0.05) compared to PBS-treated mice (Fig. 4A). In contrast, immunized male mice showed no significant differences in cytokine levels compared to PBS-treated mice (Fig. 4B). These data suggest a significant neuroinflammatory response to passive immunization in female but not male mice at 22 months old.

### TGFβ and SDF1 are negatively correlated with brain Aβ, while TNFα and MCP-1 are positively correlated

**Figure 5:**
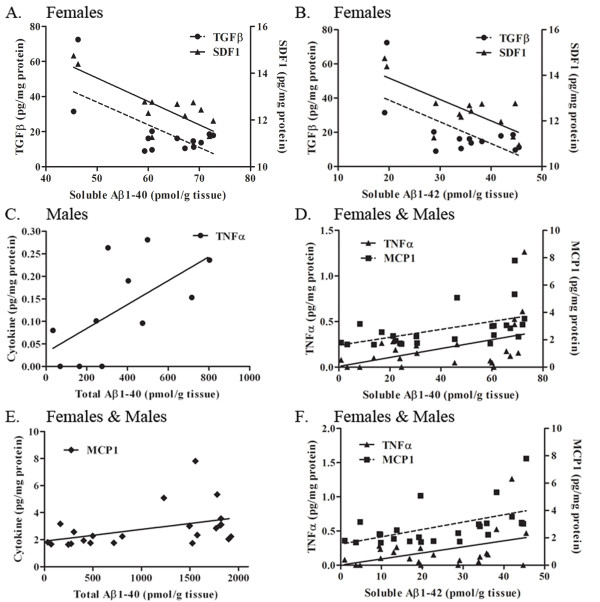
**TGFβ and SDF1 are negatively correlated with brain Aβ, while TNFα and MCP-1 are positively correlated in 22 month old mice**. A. Correlational analyses were performed between inflammatory cytokines and soluble (DEA) brain Aβ1-40 levels in female mice. TGFβ and SDF1 were significantly negatively correlated with Aβ1-40 levels (R^2 ^= 0.4633, p < 0.01; R^2 ^= 0.6421, p < 0.001, respectively). Other cytokines were not significantly correlated with soluble Aβ1-40. B. TGFβ and SDF1 were also significantly correlated with soluble Aβ1-42 levels (R^2 ^= 0.4371, p < 0.01; R^2 ^= 0.5489, p < 0.01, respectively). Other cytokines were not significantly correlated with soluble Aβ1-42. C. Correlational analyses were performed between inflammatory cytokines and total Aβ1-40 levels in male mice. TNFα was significantly correlated with total (FA) Aβ1-40 levels (R^2 ^= 0.3851, p < 0.05). Other cytokines were not significantly correlated with total Aβ1-40. D. Data from female and male mice were combined and correlational analyses performed. TNFα and MCP-1 were significantly correlated with soluble Aβ1-40 levels (R^2 ^= 0.1871, p < 0.01; R^2 ^= 0.2466, p < 0.01, respectively). Other cytokines were not significantly correlated with soluble Aβ1-40. E. MCP-1 was significantly correlated with total Aβ1-40 levels in females and males (R^2 ^= 0.1780, p < 0.05). F. TNFα and MCP-1 were significantly correlated with soluble Aβ1-42 levels in females and males (R^2 ^= 0.2018, p < 0.05).

Certain proinflammatory cytokines and chemokines are up-regulated in human AD brain, such as TNFα and IL1, while others are down-regulated. To determine whether specific CNS cytokine levels correlated with brain Aβ levels in 22 month-old female or male 3 × tg AD mice, we performed the following correlation analyses. As soluble Aβ40 or Aβ42 increased, TGFβ and SDF1 decreased in females (Fig. 5A &5B). In addition, as insoluble and total Aβ40 increased, TNFα also increased in males (data not shown, and Fig. 5C). When data from female and male mice were combined, we again found that TNFα and soluble Aβ40 and Aβ42 were positively correlated, and in addition, MCP-1 and all species of Aβ40 (soluble, insoluble, and total) and soluble Aβ42 were also positively correlated (Fig. 5D-F). Collectively, these data reveal a consistent positive correlation of brain Aβ levels with the proinflammatory cytokines TNFα and MCP-1, and a negative correlation of Aβ with TGFβ and SDF-1.

### Immunized males show a decreased trend in phospho-tau

**Figure 6:**
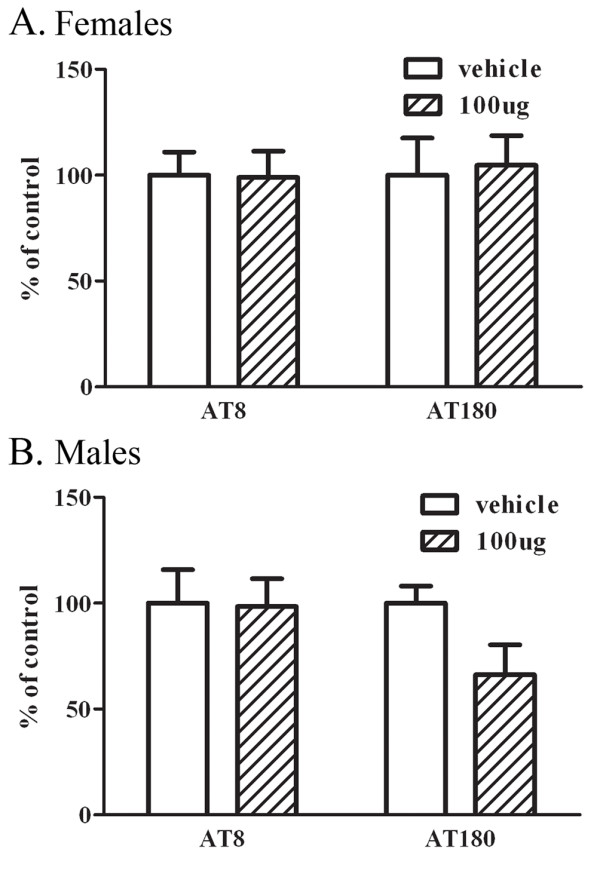
**PFA1 treated 22 month old male, but not female, mice show a decreased trend in phospho-tau**. A. Brain homogenates from PFA1-treated 22 month old female mice were Western blotted for phospho-tau (AT8, AT180) or total tau. PFA1-treated female mice did not show any significant differences in phospho-tau by either AT8 or AT180 normalized to total tau levels. B. Brain homogenates from PFA1-treated 22 month old male mice were Western blotted for phospho-tau or total tau. PFA1-treated male mice showed a trend towards decreased phospho-tau by AT180 normalized to total tau (66%) compared to vehicle-treated controls.

As demonstrated above, PFA1 immunization decreased Aβ levels in male 3 × tg AD mice, which express three mutant human transgenes - APP, PS-1, and Tau. Therefore, we determined whether PFA1 immunization also affects hTau pathology. To test this, we examined phospho-tau and total tau levels in PFA1-treated versus PBS-treated mice. We found no difference in phospho-tau normalized to total tau in immunized female mice (Fig. 6A). However, we found a decreasing but non-significant trend (18%) in phospho-tau in immunized males, suggesting that passive immunization may have a downstream beneficial effect on phospho-tau (Fig. 6B). In aggregate, these data reveal that passive immunization of 22 month old 3 × tg AD mice resulted in decreased brain Aβ and increased serum Aβ with no change in CNS inflammatory cytokines in male mice, while in female mice with greater amyloid burden we found no effect on either brain or serum Aβ, and accompanied increased CNS proinflammatory cytokine levels.

### Immunized 17 month old female mice reveal a decreasing trend in brain Aβ

**Figure 7:**
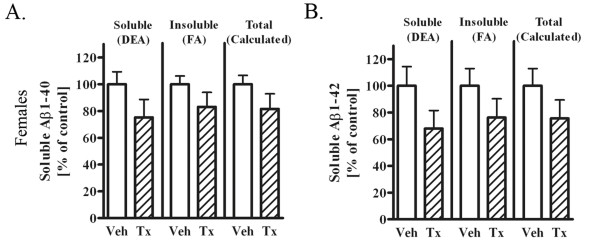
**17 month-old PFA1-treated female mice show trends towards decreased brain Aβ40 and Aβ42**. A. Aβ1-40 levels were measured from DEA-extracted (soluble proteins, left) and FA-extracted (insoluble, middle) brain lysates from vehicle (n = 5) or PFA1-treated (n = 5) 17 month-old female mice. Total Aβ levels were calculated as the sum of soluble and insoluble Aβ levels. There was a trend towards decreased soluble (25%) and insoluble (17%) Aβ1-40 levels in PFA1-treated female mice. B. Aβ1-42 levels were measured from DEA and FA lysates from the same female mice treated with vehicle or PFA1. There was a trend towards decreased soluble (32%) and insoluble (24%) Aβ1-42 levels in PFA1-treated female mice.

We wished to determine whether these results were due either to sex or to amyloid burden, as the female 3 × tg AD mice have a significantly greater amyloid load compared to male mice of the same age [18]. Therefore, we conducted identical experiments with a younger - 17 month old - cohort of female mice. We hypothesized that if the treatment was effective only in male mice due to milder burden of pathology, then younger female mice with reduced pathology may exhibit similar responses. We performed the same 4-week passive immunization treatment with PFA1 on 17 month-old female 3 × tg AD mice, and determined brain levels of soluble and insoluble Aβ40 and Aβ42, and calculated total Aβ from the sum of soluble and insoluble Aβ. Immunized 17 month old female mice revealed a trend towards decreased soluble and insoluble Aβ40 (25% soluble, 17% insoluble) and Aβ42 (32% soluble, 24% insoluble) following treatment (Fig. 7A &7B). These data suggest that disease-burden, and not sex, may be the primary factor driving discrepant results we found in 22 month old male versus female mice.

### Plasma Aβ is increased in immunized 17 month old female mice

**Figure 8:**
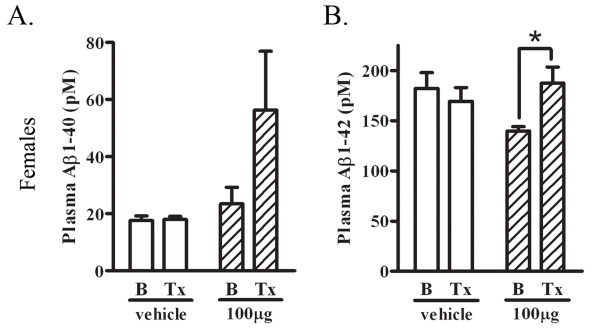
**Plasma Aβ is increased in 17 month-old PFA1-treated female mice**. A. Aβ1-40 levels were measured from plasma collected from vehicle and PFA1 treated 17 month-old female mice before and after 4 weeks of treatment. There was no significant change in plasma Aβ1-40 levels following treatment in vehicle treated animals. There was a trend towards increased Aβ1-40 levels in PFA1-treated female mice (140%) after 4 weeks of treatment. B. Aβ1-42 levels were measured from plasma collected from 17 month-old vehicle and PFA1 treated female mice before and after treatment. There were no significant changes to Aβ1-42 in vehicle-treated animals, but a significant 34% increase (p < 0.05) in PFA1-treated animals following treatment.

Next, we determined whether plasma Aβ levels were affected by immunization. We measured plasma Aβ levels from blood collected before and after the 4 week treatment paradigm. We found that PFA1-immunized 17 month old female mice revealed a significant 34% increase in Aβ42 (p < 0.05), and an increasing trend (140%) in Aβ40, following treatment (Fig. 8A &8B).

### MCP-1 is decreased, and is positively correlated with brain Aβ, in immunized 17 month old female mice

**Figure 9:**
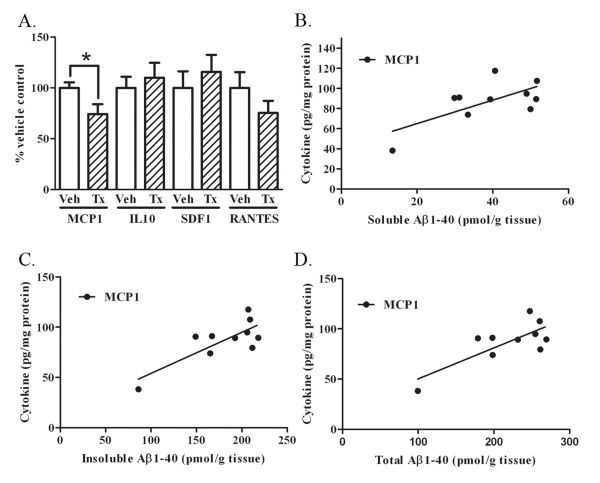
**CNS MCP-1 is decreased, and is positively correlated with brain Aβ levels, in 17 month-old female mice**. A. Levels of inflammatory cytokines MCP-1, IL10, SDF1, and RANTES were measured from brain homogenates of 17 month-old vehicle or PFA1 treated female mice. There were no significant differences in IL10, SDF1, or RANTES between vehicle and PFA1 treated groups. There was a significant 26% (p < 0.05) increase in MCP1 following PFA1 treatment in female mice. B. Correlational analyses were performed between inflammatory cytokines and soluble (DEA) brain Aβ1-40 levels in female mice. MCP1 was significantly positively correlated with soluble Aβ1-40 levels (R^2 ^= 0.4553, p < 0.05). Other cytokines were not significantly correlated with soluble Aβ1-40. C. MCP1 was also significantly correlated with insoluble Aβ1-40 levels (R^2 ^= 0.6149, p < 0.01). Other cytokines were not significantly correlated with insoluble Aβ1-40. D. MCP1 was significantly correlated with the calculated levels of total Aβ1-40 levels (R^2 ^= 0.5835, p < 0.01).

PFA1 immunization significantly increased the levels of TNFα and MCP1 in 22 month old female mice. Therefore, we determined whether cytokine levels were altered with immunization in the 17 month old group of female mice. To test this, we measured brain levels of MCP-1, IL-10, SDF-1, and RANTES in PBS-treated versus PFA1-treated groups. The immunized female mice had a significant 26% decrease in MCP-1 following treatment (p < 0.05) (Fig. 9A).

Finally, we determined whether specific CNS cytokines were consistently correlated with brain Aβ levels, and found that MCP-1 decreased as soluble, insoluble, and total Aβ40 in female mice decreased (R^2 ^= 0.45, p < 0.05; R^2 ^= 0.615, p < 0.01, R^2 ^= 0.764, p < 0.01, respectively) (Fig. 9B-D). This data is consistent with results found with the 22 month old mice.

## Discussion

Here, we examined the efficacy of a passive immunization paradigm using the monoclonal antibody PFA1 in reducing brain Aβ in a triple transgenic model of AD. We first demonstrate that the antibody binds to both Aβ40 and Aβ42 species, and binds to both oligomeric and monomeric forms (Fig. 1). Chronic passive immunization of 22 month-old 3 × tg AD mice resulted in a significant decrease in brain Aβ accompanied by an increase in serum Aβ in male mice with intermediate (less advanced) Aβ/amyloid pathology (Fig. 2, 3). However, immunization of 22 month-old female 3 × tg AD mice with advanced Aβ pathology had no effect on reducing brain Aβ while increasing levels of inflammatory cytokines TNFα and MCP-1 (Fig. 2, 3, 4). These effects were presumably pathology burden- and not sex-dependent, as immunization of 17 month-old female mice with intermediate pathology resulted in trends towards decreased brain Aβ, increased clearance of Aβ to the periphery, and a decrease in the inflammatory cytokine MCP-1 (Figs. 7, 8, 9).

We demonstrated a significant reduction in total Aβ42 in immunized 22 month-old male 3 × tg AD mice. Although effects on total Aβ40 or soluble Aβ40 or Aβ42 were not statistically different, all species of Aβ showed a consistent trend towards decreased Aβ following passive immunization. Similarly, 17 month-old female 3 × tg AD mice showed consistent, but non-significant, trends toward decreased Aβ of all forms. These results were in contrast to those observed in the 22 month-old females, where there was no evidence of decreased Aβ of any species, with a possible increase in soluble Aβ42. While our results suggest that there may be an effect of PFA1 passive immunization on reducing all forms of brain Aβ at intermediate pathological stages, it will be necessary to expand the size and scope of this study due to address this issue.

The peripheral sink hypothesis of Aβ clearance suggests that increasing Aβ-binding agents in the periphery enhances Aβ efflux from the brain [14,22]. In our studies, we observed a concurrent increase in serum Aβ with a decrease in brain Aβ in 22 month-old male mice, and a concurrent increase in serum Aβ with a trend towards decreased brain Aβ in 17 month-old female mice. Our findings support the possibility that both passive and active immunization involve antibody-mediated clearance of Aβ from the brain to the periphery. This effect can occur independently of an antibody effect in the brain which may incite microglial phagocytosis. Aβ-binding agents, not limited to antibodies, such as heparin [23] and the Nogo receptor [24], are also effective in reducing brain Aβ. In addition, active immunization of an AD mouse model lacking the FcR, which is necessary for immune activation, still reduces brain Aβ [25], and deglycosylated antibodies, which do not bind the FcR, increase Aβ clearance to the periphery [26]. These findings suggest that peripheral clearance of Aβ is a viable mechanism by which passive or active immunization may decrease brain Aβ levels.

Although passive immunization does not directly involve T-cell activation, it can still induce microglial activation and upregulation of inflammatory cytokines. Indeed, we observed that the inflammatory cytokine TNFα and chemokine MCP-1 were increased following immunization in 22 month-old female mice in the absence of any benefit on lowering brain Aβ. TNFα is increased in human AD brains [27] and is increased following antibody treatment in cultured cells [26]. MCP-1 is a chemokine which is also upregulated in serum of AD patients and may play a role in early inflammatory events [28]. The effects of microglial activation may be pathology-dependent, as TNFα induction in younger PS1xAPP mice results in microglial phagocytosis while activation at older ages results in cytotoxic effects [29]. Thus, immune modulation at advanced pathology may increase potentially adverse inflammatory effects of microglial activation compared to a neuroprotective phagocytic Aβ/amyloid clearance effect found with lower disease burden.

In addition to the increased cytokine levels following passive immunization in mice with advanced pathology, we also observed significant positive correlations between increasing Aβ levels and increasing TNFα and MCP-1 levels, further supporting a pro-inflammatory role for these cytokines. In contrast, we observed an inverse correlation with other cytokines -- as Aβ levels increased, TGFβ and SDF1 decreased, implicating a beneficial role for these cytokines in mediating anti-Aβ effects. This hypothesis is supported by evidence that overexpressing TGFβ in hAPP mice results in decreased numbers of plaques [30] and reducing TGFβ signaling in mice results in increased Aβ accumulation and neurodegeneration [31]. Expression of the chemokine SDF1 is decreased in early AD brains [32], suggesting that disregulation of its chemotactic signaling properties may either be an early symptom of AD or may contribute to later AD pathology.

## Conclusions

In conclusion, our study is the first to establish pathology burden-dependent effects of passive immunization in an animal model of AD. Treatment at an intermediate pathological stage is more effective at reducing brain Aβ, while treatment at a more advanced stage induces neuroinflammatory effects. In addition, our findings support previous studies that passive immunization, as well as active immunization, results in the clearance of Aβ from the brain to the periphery. High levels of Aβ pathology may contribute to neurotoxic effects mediated by microglial inflammation, while immunomodulation at an intermediate stage may allow the larger pool of soluble Aβ to be sequestered into the periphery. These findings highlight the importance of considering disease burden in determining optimal passive immune timing and dosage in order to maximize therapeutic effects and minimize adverse effects when considering a human intervention.

## Abbreviations used

APP: amyloid precursor protein; AD: Alzheimer's disease; DEA: diethylamine; FA: formic acid; IL1β: interleukin 1β; TGFβ: transforming growth factor β; TNFα: tumor necrosis factor α; MCP1: monocyte chemotactic protein 1; SDF1: stromal cell-derived factor 1; CCL5: chemokine ligand 5, RANTES

## Competing interests

The authors declare that they have no competing interests.

## Authors' contributions

SSM, ES, SA, MS, TN, JSP, and RW conducted experiments. SSM, HSH, and RST prepared the manuscript. CD, YM and RST conceived the project and designed experiments. IM, FB, and GWR provided valuable scientific input, and RST supervised the overall project. All authors have read and approved the final manuscript.
